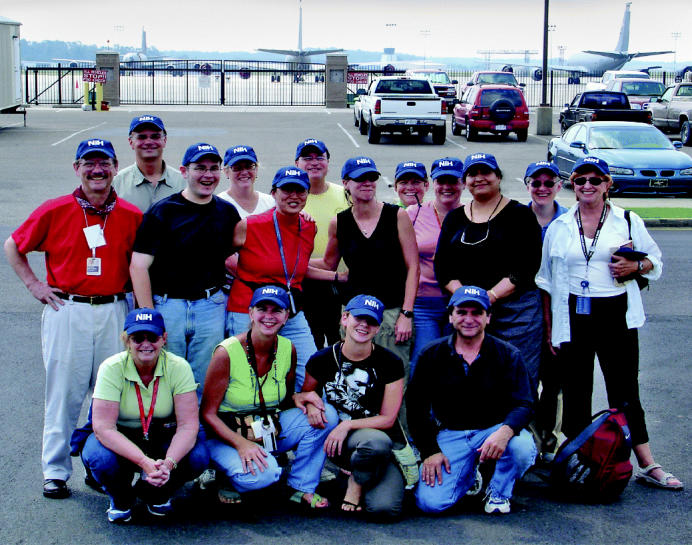# The NIEHS Responds to Hurricane Katrina

**DOI:** 10.1289/ehp.113-a722

**Published:** 2005-11

**Authors:** David A. Schwartz

**Affiliations:** Director, NIEHS and NTP, david.schwartz@niehs.nih.gov

I am very proud of the role that the NIEHS has played in the Hurricane Katrina relief efforts. Extramural and intramural scientists have jointly developed and are implementing both short-term and long-term responses to this natural disaster. Many individuals have contributed to our response, but I especially want to acknowledge the leadership of Sam Wilson, Rich Freed, Bill Suk, Allen Dearry, Mary Wolfe, and Angie Sanders. These six people have played a critical role in responding to the devastation and hardship that was caused by Hurricane Katrina. While these people deserve special recognition, I am overwhelmed by the outpouring of support, encouragement, and volunteerism from many other members of our institute.

Here at home, Bill Suk continues to lead an effort to develop an NIEHS website that will utilize geographic information system (GIS) mapping to help identify the environmental hazards from Katrina that may affect the health of those living in or returning to the Gulf region. This website will be continuously updated through a collaboration established between the NIEHS and the University of California, San Diego (UCSD), super-computing center. Mark Ellisman at UCSD and Marie Lynn Miranda at Duke University have played key roles in developing this website. We anticipate that the website will serve as a national resource to track environmental hazards and focus various medical and environmental responses in areas that are in greatest need. Included on this website is up-to-date information on safety and hazardous waste cleanup training for the thousands of workers involved in the cleanup and recovery activities in the region, as well as a compendium of environmental health resources for medical responders.

Allen Dearry has established a consultative service to address environmental hazards that may have an impact on human health. This consultative service, available through the Katrina website, is linked to experts in our intramural and extramural research programs who have provided informed responses to specific environmental health concerns of residents, healthcare providers, and workers involved in the rescue and recovery efforts.

We have established a cooperative relationship with the Centers for Disease Control and Prevention (CDC) to assist them in the deployment of public health teams to areas that are in need of food, shelter, and a safe water supply. Mary Wolfe has taken a leadership role in this effort and is working directly with Tom Sinks of the CDC to provide expertise from the NIEHS to support the CDC in more effectively responding to the emerging public health needs.

The NIH deployed a team to the Gulf region to provide direct medical care to the victims of Hurricane Katrina. The NIEHS played a major role in this effort, and established a North Carolina contingent that consisted of physicians, nurses, and other health care providers from both the NIEHS and Duke University. Sam Wilson and Rich Freed coordinated activities between North Carolina and the central headquarters at the National Institutes of Health (NIH). I was fortunate to be one of the members of the team that traveled to Meridian, Mississippi, and established a 500-bed medical facility there including a triage area, ambulatory care area, acute care component, and an area for hospitalized patients.

Unfortunately, many of those affected by Katrina were reluctant to leave their homes for medical care despite the extensive amount of local destruction. Consequently, individuals in our group made several trips to the Gulfport coastal region, an area most severely affected by the hurricane, to assess the extent of medical need and to determine whether we could safely establish a medical facility in that region. As all of you know, the extent of destruction was overwhelming, with cars upturned, tractor trailers scattered like matchsticks, homes completely leveled, buildings destroyed, and rotting food scattered throughout the area; the Gulf Coast was nothing short of what one would expect in a war zone.

An accelerated environmental health research plan is vital to protecting vulnerable populations during present and future catastrophes.

Despite this, we found that the medical infrastructure was relatively intact. Patients were being seen effectively in clinics, and hospitals were open with sufficient bed capacity. We visited at least six different communities in the Gulfport region, and were sufficiently convinced that our team of health care providers was simply not needed. Thus, we decided to suspend the NIH medical mission, although we remain on call if other needs are identified. I was impressed with the cooperative and constructive relationship that was established between the NIH, Duke University, and the Public Health Service. Moreover, other universities, including the University of North Carolina at Chapel Hill, are providing additional support to the region.

Hurricane Katrina has made it abundantly clear that there is an unmet need for a national coordinated response plan to assess environmental and biological exposures to hazardous agents, to understand the relationship of exposures to adverse health outcomes through appropriate surveillance, and to develop early prevention and intervention strategies designed to identify at-risk individuals and reduce morbidity and mortality. To address this need, the NIEHS is working with the CDC and the EPA to develop a proposal to prevent adverse health consequences related to environmental conditions and exposures in the wake of Hurricane Katrina, and to establish the scientific expertise and infrastructure in environmental health needed to address both immediate and future preventive health care needs and the response capacity following man-made and natural disasters. We believe that an accelerated environmental health research plan is vital to protecting vulnerable populations during present and future catastrophes.

In tough times, the strengths and weaknesses of an institute become apparent. I’m proud to say that in the time since Hurricane Katrina struck, the NIEHS has proven to be an exceedingly strong institution with vitality, resolve, and a deep sense of dedication to relieving human suffering. I’m sincerely proud to be a member of the NIEHS, and I am confident that our team is fully capable of addressing the unforeseen challenges and opportunities that lie ahead.

## Figures and Tables

**Figure:**